# Blockade of mIL‐6R alleviated lipopolysaccharide‐induced systemic inflammatory response syndrome by suppressing NF‐κB‐mediated Ccl2 expression and inflammasome activation

**DOI:** 10.1002/mco2.132

**Published:** 2022-05-06

**Authors:** Ji‐Min Dai, Xue‐Qin Zhang, Jia‐Jia Zhang, Wei‐Jie Yang, Xiang‐Min Yang, Huijie Bian, Zhi‐Nan Chen

**Affiliations:** ^1^ National Translational Science Center for Molecular Medicine&Department of Cell Biology State Key Laboratory of Cancer Biology the Fourth Military Medical University Xi'an P.R. China; ^2^ Faculty of Hepato‐Biliary‐Pancreatic Surgery The First Medical Center of Chinese People's Liberation Army (PLA) General Hospital Beijing P.R. China

**Keywords:** Ccl2, mIL‐6R, monoclonal antibody, NF‐κB, pyroptosis, SIRS

## Abstract

Systemic inflammatory response syndrome (SIRS) is characterized by dysregulated cytokine release, immune responses and is associated with organ dysfunction. IL‐6R blockade indicates promising therapeutic effects in cytokine release storm but still remains unknown in SIRS. To address the issue, we generated the human *il‐6r* knock‐in mice and a defined epitope murine anti‐human membrane‐bound IL‐6R (mIL‐6R) mAb named h‐mIL‐6R mAb. We found that the h‐mIL‐6R and the commercial IL‐6R mAb Tocilizumab significantly improved the survival rate, reduced the levels of TNF‐α, IL‐6, IL‐1β, IFN‐γ, transaminases and blood urea nitrogen of LPS‐induced SIRS mice. Besides, the h‐mIL‐6R mAb could also dramatically reduce the levels of inflammatory cytokines in LPS‐treated THP‐1 cells in vitro. RNA‐seq analysis indicated that the h‐mIL‐6R mAb could regulate LPS‐induced activation of NF‐κB/Ccl2 and NOD‐like receptor signaling pathways. Furthermore, we found that the h‐mIL‐6R mAb could forwardly inhibit Ccl2 expression and NLRP3‐mediated pyroptosis by suppressing NF‐κB in combination with the NF‐κB inhibitor. Collectively, mIL‐6R mAbs suppressed NF‐κB/Ccl2 signaling and inflammasome activation. IL‐6R mAbs are potential alternative therapeutics for suppressing excessive cytokine release, over‐activated inflammatory responses and alleviating organ injuries in SIRS.

## Graphical abstract

SIRS is characterized by dysregulated cytokine release and inflammatory responses. IL‐6R mAbs like Tocilizumab displays therapeutic effects in cytokine release storm and inflammatory diseases but remains unknown in SIRS. To address the issue, we generated the human *il‐6r* knock‐in mice and a defined epitope murine anti‐human membrane IL‐6R (mIL‐6R) monoclonal antibody named h‐mIL‐6R mAb. We found that the h‐mIL‐6R mAb and Tocilizumab alleviated LPS‐induced SIRS in the transgenic mice. Besides, the h‐mIL‐6R mAb could also inhibit Ccl2 expression and NLRP3‐mediated pyroptosis by suppressing the NF‐κB signaling pathway. This study indicated that mIL‐6R mAbs suppressed NF‐κB/Ccl2 signaling and inflammasome activation in LPS‐induced inflammatory responses and SIRS.

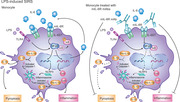

## 1 INTRODUCTION

Systemic inflammatory response syndrome (SIRS) is a severe and almost uncontrollable pathological condition with excessive cytokine release throughout the body.^1^ With high morbidity and high mortality, SIRS accounts for up to 30% of the hospital patients and even no less than half of intensive care unit patients.^2^ Furthermore, SIRS is pathologically related to sepsis, the syndrome which can lead to multiple organ dysfunction and even life‐threatening organ failure.^3^ It is estimated that global annual morbidity of sepsis is 31.5 million cases and mortality is 5.3 million deaths.^2^ Therefore, it is imperative to effectively intervene with SIRS to prevent poor prognosis. As triggers of SIRS, trauma and infection provoke host defensive responses and herein, interleukin‐6 (IL‐6) is rapidly synthesized and forwardly promotes the host defense.^4^ It has been demonstrated that increased serum IL‐6 concentration reflects SIRS state and predicts adverse events of SIRS patients, suggesting the important role of IL‐6 elevation in SIRS.^5^ More importantly, excessive production of IL‐6 and activation of IL‐6 signaling contribute to cytokine‐release syndrome (CRS) and hyperinflammatory state but also synergize anti‐inflammatory cytokines to develop immunosuppression.^6^ This counter‐regulating suppressive state toward SIRS is termed as compensatory anti‐inflammatory response syndrome (CARS) which is attributed to the risk of infectious complications and poor prognosis in SIRS patients.^7^ Therefore, blockade of IL‐6 signaling is regarded as the effective strategy for management of SIRS.

IL‐6 signaling is mediated by IL‐6R complex which consists of IL‐6R (also known as IL‐6RA) and IL‐6 signaling transducer (IL‐6ST, also known as gp130 and IL‐6RB).^8^ IL‐6R is the mandatory element for IL‐6/IL‐6R complex formation, and this process is prerequisite for IL‐6 signaling transduction mediated by IL‐6ST. The IL‐6R gene was mapped on the long arm of chromosome 1 in the q21.3 cytogenetic band. Human IL‐6R mainly consists of the Ig‐like C2‐type domain (26‐112 amino acids/aa), the fibronectin type‐III 1 domain (113‐217aa) and the fibronectin type‐III 2 domain (218‐316aa) followed by the flexible so‐called stalk region, a transmembrane and a short intracellular region. The two fibronectin type‐III like domains are extracellular part of IL‐6R and form the cytokine‐binding domain. More importantly, IL‐6R exists in a transmembrane form (membrane‐bound IL‐6R, mIL‐6R) and a soluble form (sIL‐6R) which can be generated by cleavage of mIL‐6R or translation of alternatively spliced *il‐6r* mRNA.^9^ Moreover, IL‐6R functions as the binding subunit for IL‐6 and its activation induces acute‐phase reactions, immune response modulation, and hematopoiesis.^10^ Furthermore, IL‐6R initiates IL‐6 signaling in three different modes after IL‐6 binding. Generally, mIL‐6R induces classic signaling, which is associated with acute‐phase response, humoral immunity, etc.^11^ sIL‐6R mediates trans‐signaling involved in pro‐inflammatory process like fever.^12^ Moreover, trans‐presentation by mIL‐6R is concerned with specialized intercellular communication which primes T helper 17 (Th17) cell commitment.^13^ These modes of IL‐6 signaling activate Janus kinase (JAK)/mitogen‐activated protein kinase (MAPK) and JAK/signal transducer and activator of transcription 3 (STAT3) signaling pathways which regulate inflammatory and immune responses. Identification of these IL‐6R recognition‐dependent signaling modes sheds light on the importance of mIL‐6R in inflammatory responses and immunomodulation. Actually, IL‐6R blockade has become the novel strategy to treat inflammatory and immune disorders characterized by IL‐6 overproduction. For instance, the humanized anti‐IL‐6R monoclonal antibody (mAb) Tocilizumab has been proved with remarkable efficacy for the treatment of rheumatoid arthritis, Castleman disease, and juvenile idiopathic arthritis.^8^ More importantly, IL‐6R blockade also displays therapeutic effects for acute and intractable inflammatory diseases such as CRS and SIRS.^8^ It has been revealed that mIL‐6R could mediate sustained production of IL‐6 by a positive feedback loop through activated Src kinase and MAPK signaling pathways.^14^ The mIL‐6R‐mediated IL‐6 recognition and subsequent production of IL‐6 magnifies the pro‐inflammatory effects of IL‐6 in the SIRS process, which indicates that mIL‐6R plays an important role in SIRS.^15^ However, how mIL‐6R blockade alleviates immune responses in SIRS is still far from understood.

Nuclear factor kappa B (NF‐κB) is the transcription factor specialized for cellular rapid response to stimuli including lipopolysaccharide (LPS), tumor necrosis factor‐α (TNF‐α), IL‐1β and is involved in pathogenesis of SIRS.^16^ Activation of NF‐κB signaling in innate immune cells such as monocyte and macrophage leads to increased expression of various pro‐inflammatory mediators including cytokines (such as TNFs, IL‐6, etc.), chemokines (such as monocyte chemotactic protein‐1, MCP‐1), adhesion molecules (such as E‐selectin), and enzymes (such as cyclooxygenase‐2), which remarkably prompts the onset of SIRS.^17^ Of note, IL‐6 signaling activation forwardly promotes activation of NF‐κB and magnifies the inflammatory response which forms a positive feedback loop.^18^ In contrast, it has been documented that IL‐6R blockade mediated by Tocilizumab significantly attenuated organ dysfunction and improved prognosis of SIRS/sepsis via NF‐κB inhibition.^19^ Among the downstream regulated genes, C–C motif chemokine 2 (Ccl2, also known as MCP‐1) has been found to be activated by NF‐κB in the context of inflammatory responses including SIRS and sepsis.^20^ Ccl2 elevation has been reported in peripheral circulation of sepsis patients with gram‐negative infection.^21^ Moreover, the expression of Ccl2 has been found to increase in monocyte/macrophage from lesions of atherosclerosis and rheumatoid arthritis patients, indicating that Ccl2 is responsible for inflammatory cell infiltration and tissue destruction.^22^ Nevertheless, it is unknown whether mIL‐6R blockade‐mediated NF‐κB inhibition reduces excessive release of Ccl2 thereby alleviating inflammatory responses in SIRS.

Furthermore, NF‐κB signaling can also facilitate inflammasome‐mediated pyroptosis following the activation by pattern recognition receptors such as Toll‐like receptor 4 (TLR4), TNFR, and IL‐1R. It consequently promotes synthesis of pro‐IL‐1β and NLR pyrin domain‐containing protein 3 (NLRP3).^23^ Previous studies have revealed that NLRP3 inflammasome activation is responsible for pyroptosis of Kupffer cells, hepatic endothelial cells, and lung endothelial cells in SIRS/sepsis, which is associated with multiple organ dysfunctions and deteriorated prognosis.^24^, ^25^, ^26^ Besides, NF‐κB signaling‐mediated TNF has been reported to prime NLRP3 for assembly and activation.^27^ Hence, it is reasonable to speculate that NF‐κB signaling induces Ccl2 and NLRP3 in the context of SIRS and both of them bring about enhanced inflammatory responses and injury. However, it remains unknown whether mIL‐6R blockade decreases NLRP3 inflammasome via inhibiting NF‐κB signaling in SIRS.

In this study, we generated IL‐6R transgenic mice (IL6r‐e(hIL6R)1) with human *il‐6r* gene knock‐in in replacement of the murine *il‐6r* gene by the CRISPR‐Cas9 technology. Besides, we designed and produced a murine mAb‐specific targeting human mIL‐6R (h‐mIL‐6R mAb) via hybridoma technology. After identification of the transgenic mice and the h‐mIL‐6R mAb, we constructed LPS‐induced murine SIRS model and then treated them with the h‐mIL‐6R mAb or Tocilizumab. We found that both the h‐mIL‐6R mAb and Tocilizumab (IL‐6R blockades) significantly reduced elevation of pro‐inflammatory cytokines, alleviated organ injury and improved mortality in the LPS‐induced SIRS model. We isolated the peripheral blood mononuclear cell (PBMC) from the SIRS model and employed RNA‐seq analysis which indicated that the h‐mIL‐6R mAb treatment could regulate Ccl2 expression, NOD‐like receptor signaling pathway, and TNF signaling pathway. To verify the underlying mechanism, we used human monocyte cell line THP‐1 and found that the h‐mIL‐6R mAb not only effectively suppressed JAK/STAT3 and JAK/MAPK signaling pathways downstream of mIL‐6R, but also protected LPS‐treated THP‐1 cells from inflammatory responses via inhibiting NF‐κB/Ccl2, as well as pyroptosis via inhibiting NF‐κB/NLRP3/Gasdermin D (GSDMD) signaling pathway. Collectively, this study indicated that the IL‐6R blockade relieved SIRS in the murine model, potentially by protecting monocyte from NF‐κB‐activated inflammatory signaling and pyroptosis.

## 2 RESULTS

### 2.1 Generation and identification of human *il‐6r* knock‐in mice

To explore the effects of mIL‐6R‐mediated signaling and mIL‐6R blockade on SIRS, we employed CRISPR‐Cas9 technology to knock in the human *il‐6r* gene because it is responsible for mIL‐6R expression (Figure S1A). In brief, we designed guide RNA (gRNA) for *il‐6r* as GAAGGAAGCATGCTGACCGT and performed nucleic acid electrophoresis to verify the validity of the *il‐6r* gRNA (Figure S1B), as well as the T7‐Cas9 vectors and Cas9 mRNA (Figure S1C). We constructed the homologous recombinant plasmid and identified the plasmid by PCR (Figure S1D). The nucleic acid electrophoresis showed the three spliced fragments of the homologous recombinant plasmid, with length of 852 bp, 6665 bp, and 8137 bp, respectively (Figure S1E). The homologous recombination mice named IL6r‐e(hIL6R)1 were confirmed by PCR (Figure S1F). The amplification products showed that in 3′ homology arm of homologous recombinant positive mice were expected to be a 5.5 kbp‐long fragment while that of the homologous recombinant negative mice were expected to yield no fragment. The red lines indicated that the fragments of targeted allele PCR products were sequenced for comparing with the corresponding human *il‐6r* gene DNA sequence. The electrophoresis of 5′ homology arm products showed that within the 40 P_0_ transgenic mice, samples of No. 3, 8, 11, 14, and 23 mice were amplified with expected products of 5.6 kbp‐long (Figure S1G). Thereafter, we chose these homologous recombinant positive (5′ homology arm) mice and verified that the 3′ homology arm products were 5.5 kbp, consistent with the expected length by electrophoresis (Figure S1H). Besides, the sequencing of PCR products confirmed that there were no mutation or insertion/deletion of DNA sequences in transgenic mice (data not shown).

**FIGURE 1:**
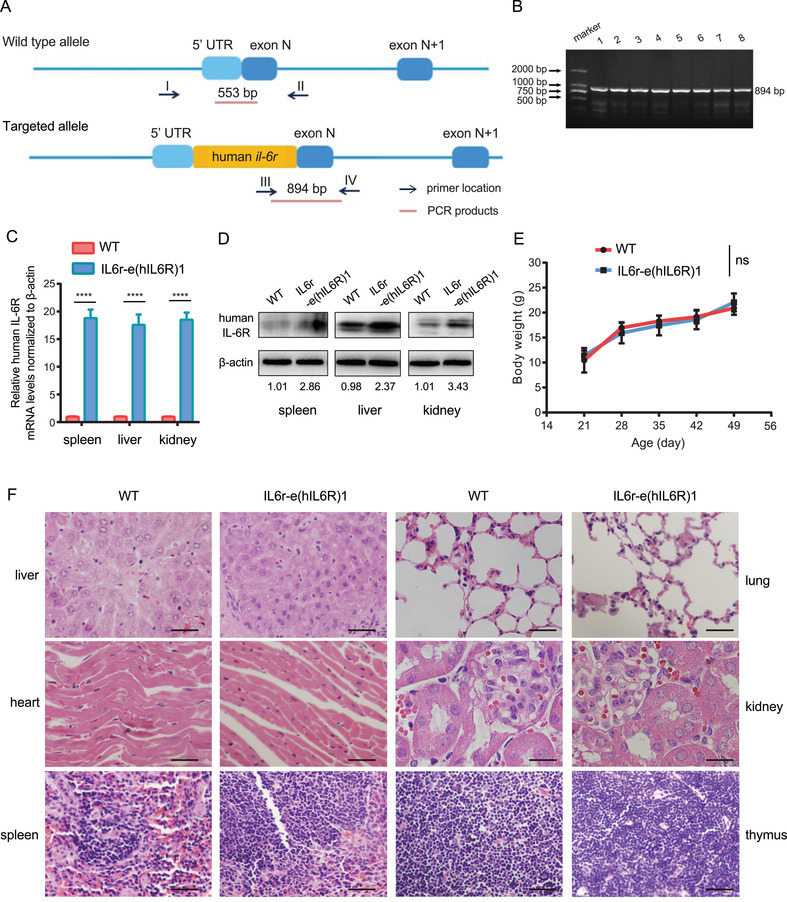
Identification and phenotype analysis of human *il‐6r* gene knock‐in mice. (A) Schematic of identification for the IL6r‐e(hIL6R)1 offspring. The PCR product of the WT allele was 553 bp, while the PCR product of human *il‐6r* knock‐in allele was 894 bp. (B) Nucleic acid electrophoresis results of the P_1_ transgenic mice. (C) Relative human IL‐6R mRNA levels in spleen, liver, and kidney in IL6r‐e(hIL6R)1 mice compared with those in the WT mice measured by qRT‐PCR (*n* = 4). (D) Immunoblotting analysis of human IL‐6R expression in spleen, liver and kidney in IL6r‐e(hIL6R)1 mice compared with the WT mice. The mean of densitometry analysis was presented below the immunoblotting results (*n* = 4). (E) The curves illustrating body weight of IL6r‐e(hIL6R)1 mice between 21–49 days compared with the WT mice (*n* = 6). (F) Representative H&E staining images of the indicated organs from IL6r‐e(hIL6R)1 mice compared with those of the WT mice. Scale bar = 25 μm. Magnification, 400 × . The results were presented as mean ± SD through three independent experiments and the results were analyzed by two‐tailed Student's *t* test. **** *p *< 0.0001. ns, no significance

All the offspring generations of IL6r‐e(hIL6R)1 transgenic mice were confirmed by PCR. The PCR product of the wild‐type (WT) mice was 553 bp, while the PCR product from the homozygous offspring generations of IL6r‐e(hIL6R)1 mice was 894 bp (Figure 1A,B). Real‐time quantitative PCR (qRT‐PCR) and immunoblotting analysis showed that human *il‐6r* gene was significantly expressed in the transgenic mice compared to the WT mice on both mRNA and protein levels (Figure 1C,D). Moreover, there were no differences in dietary habits and weight growing rate between the IL6r‐e(hIL6R)1 mice and the WT mice (Figure 1E). Additionally, we performed H&E staining of the major organs including liver, heart, spleen, lung, kidney, and thymus and there were no development deficiencies in the human *il‐6r* gene knock‐in mice (Figure 1F). Therefore, human *il‐6r* knock‐in transgenic mouse IL6r‐e(hIL6R)1 was generated and available for further studies.

### 2.2 Preparation of the specific monoclonal antibodies against human mIL‐6R

**FIGURE 2:**
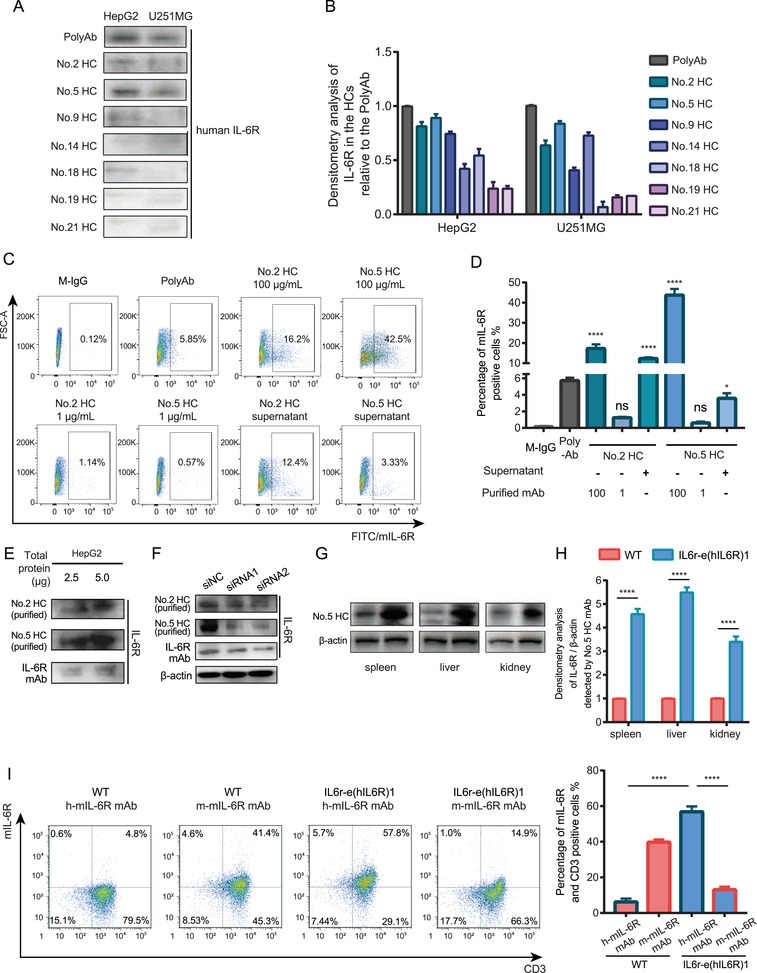
Preparation and selection of the monoclonal antibodies against human mIL‐6R. (A‐B) Immunoblotting and densitometry analysis of human IL‐6R expression in HepG2 and U251MG cell lines using the supernatants of the HCs as the primary antibody. The concentration of the total protein in the supernatants was 100 μg/ml. The PolyAb was the serum of the immunized mice, which included the mAbs against the fragment Peptide3 (sequence: EWGPRSTPSLTTKAC) of human mIL‐6R. (C,D) Representative flow cytometry images and statistical analysis of mIL‐6R expression in HepG2 cell line using the indicated antibodies as the primary antibody for flow cytometry analysis. The total protein concentration of the supernatants was 100 μg/ml. The negative control (NC) group was treated by mouse IgG (M‐IgG) (100 μg/ml). The results were compared with the NC group. (E) Immunoblotting of human IL‐6R expression in HepG2 cell line with indicated total protein quantity (2.5, 5.0 μg) by the purified No.2 and No.5 HC mAbs (100 μg/ml) and the commercialized mAb as the primary antibody. (F) Immunoblotting of human IL‐6R expression in HepG2 cell line with IL‐6R knock‐down by two different siRNAs, using the purified No.2 HC and No.5 HC mAbs (100 μg/ml) and the commercialized mAb as the primary antibody. (G,H) Immunoblotting and densitometry analysis of human IL‐6R expression in the IL6r‐e(hIL6R)1 mice using the No.5 HC mAb as the primary antibody (100 μg/ml). (I) Representative flow cytometry images and statistical analysis of human mIL‐6R expression in CD3 positive cells of PBMC from the WT mice and the IL6r‐e(hIL6R)1 mice. The No.5 HC mAb (h‐mIL‐6R mAb, 100 μg/ml) and the commercial mouse IL‐6R antibody (m‐mIL‐6R mAb) were used as the primary antibody to detect human and mouse mIL‐6R expression. The results were presented as mean ± SD through three independent experiments. The results were analyzed by one‐way ANOVA. * *p *< 0.05, ** *p *< 0.01, *** *p *< 0.001, **** *p *< 0.0001; ns, no significance

To study the effects of mIL‐6R blockade in human *il‐6r* knock‐in mice IL6r‐e(hIL6R)1, we generated 21 in‐house hybridoma clones (HCs) against the fragment Peptide3 (sequence: EWGPRSTPSLTTKAC) of human mIL‐6R by the hybridoma technology. We collected and screened all the supernatants of the cell clones to determine the specificity of the obtained clones by Peptide3‐conjugated Keyhole Limpet Hemocyanin (KLH) based enzyme‐linked immunosorbent assay (ELISA, data not shown). We selected seven HCs with relative high specificity and then, we utilized the supernatants of the seven HCs (total protein concentration 100 μg/ml) and the serum of the immunized mice (PolyAb) containing the potential human mIL‐6R mAbs as the primary antibody of immunoblotting for human mIL‐6R detection in the human mIL‐6R expressing cell line HepG2 and U251MG (Figure 2A,B). The results indicated that mAbs produced by No.2 HC and No.5 HC had relative higher specificity of human mIL‐6R among the obtained HCs. Therefore, we purified these two clones to get 100 and 1 μg/ml mAbs respectively and performed flow cytometry analysis using the purified mAbs and the supernatants of No.2 HC and No.5 HC as the primary antibody to further examine the specificity to human mIL‐6R in HepG2 cell line. We found that the percentage of the positive HepG2 cells detected by the No.2 HC and No.5 HC was significantly higher than that detected by the M‐IgG as the primary antibody (Figure 2C,D), indicating that the purified No.2 HC and No.5 HC mAbs were specific to human mIL‐6R than the M‐IgG. Besides, we performed immunoblotting with the small quantity of total protein (2.5 and 5.0 μg) from the HepG2 cell lysates and used the purified mAbs from No.2 HC and No.5 HC and the commercial IL‐6R antibody as the primary antibody of immunoblotting analysis. The results showed that the purified mAbs were able to detect mIL‐6R from the small quantity of total protein (Figure 2E). To confirm the specificity of the purified mAbs, we knocked down the expression of IL‐6R in HepG2 cells by small interfering RNAs (siRNAs) and detected the decreased IL‐6R expression level by immunoblotting analysis using the purified mAbs from the HCs as the primary antibody (Figure 2F). The results showed that the mAb from No.5 HC had relative high specificity against human mIL‐6R compared with other HCs. More importantly, the binding affinity of the mIL‐6R mAb from No.5 HC was determined by surface plasmon resonance (SPR) with the *K_d_* value of 1.1 × 10^−9^ M using the corresponding immunogen IL‐6R‐KLH. Furthermore, to verify the effectiveness of the human mIL‐6R mAb from the No.5 HC and human mIL‐6R expression in IL6r‐e(hIL6R)1 mice, we performed immunoblotting analysis using the mIL‐6R mAb from No.5 HC to detect mIL‐6R protein levels in spleen, liver, and kidney tissues from the IL6r‐e(hIL6R)1 mice (Figure 2G,H). In consistence with the results detected by the commercial human IL‐6R antibody, the expression of human mIL‐6R was significantly higher in the organs from the IL6r‐e(hIL6R)1 mice compared to the organs from the WT mice. In addition, we collected PBMC from the IL6r‐e(hIL6R)1 mice and performed flow cytometry assay using the mIL‐6R mAb from the No.5 HC to detect human mIL‐6R expression in CD3 positive cells from the PBMC. Consistently, we found that human mIL‐6R expression was significantly higher than mouse mIL‐6R expression in CD3 positive cells of the IL6r‐e(hIL6R)1 mice and more importantly, we found that the No.5 HC mAb was capable of binding mIL‐6R because human mIL‐6R expression in CD3 positive cells was significantly higher in the IL6r‐e(hIL6R)1 mice compared with that in the WT mice (Figure 2I). Taken together, after functional and structural identification, we chose the mAb from the No.5 HC (named as h‐mIL‐6R mAb) as the effective mAb against human mIL‐6R for further studies.

### 2.3 The h‐mIL‐6R mAb alleviated LPS‐induced SIRS in the IL6r‐e(hIL6R)1 mice

**FIGURE 3:**
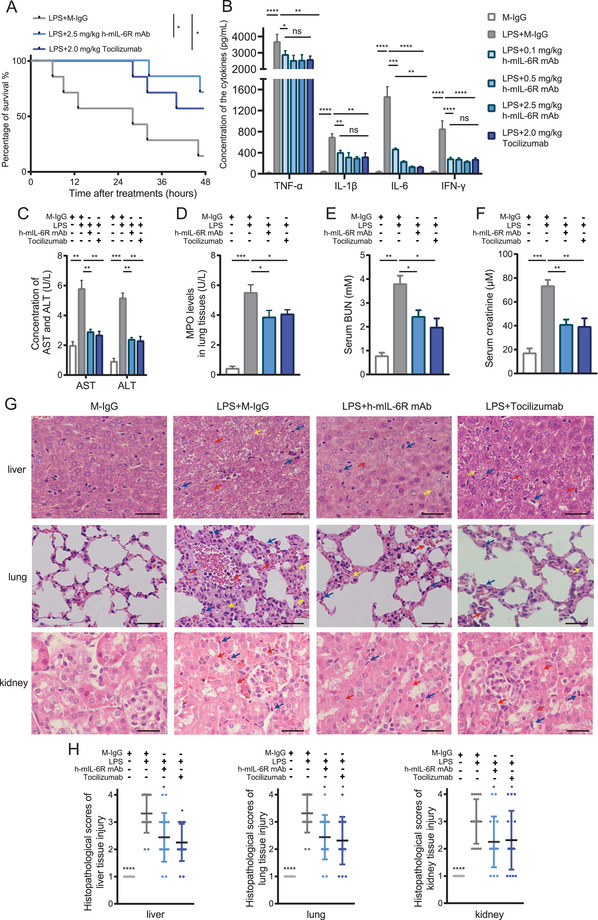
Evaluation for therapeutic effectiveness of the h‐mIL‐6R mAb and Tocilizumab for SIRS in the IL6r‐e(hIL6R)1 mice. (A) Survival curve comparison within 48 h after the indicated treatments (i.p.) in the IL6r‐e(hIL6R)1 mice: mouse IgG (M‐IgG) (2.5 mg/kg), LPS (40 mg/kg), h‐mIL‐6R mAb (2.5 mg/kg) and Tocilizumab (2.0 mg/kg) (*n* = 7). (B) Concentrations of the inflammatory cytokines including TNF‐α, IL‐1β, IL‐6, and IFN‐γ in the serum at 6 h after the indicated treatments (i.p.) detected by ELISA (*n* = 4). (C) Concentrations of liver injury biomarkers including AST and ALT in the serum at 24 h after the indicated treatments (i.p.) in the IL6r‐e(hIL6R)1 mice detected by ELISA (*n* = 4). (D) Detection of the inflammatory biomarker MPO in lung tissues at 12 h after the treatments (i.p.) in the IL6r‐e(hIL6R)1 mice (*n* = 4). (E,F) Renal function evaluated by BUN and creatinine levels in the serum at 12 h after the treatments (i.p.) in the IL6r‐e(hIL6R)1 mice (*n* = 4). (G,H) Representative H&E staining images of liver, lung, and kidney and statistical analysis of the scorings 24 h after the treatments (i.p.) in the IL6r‐e(hIL6R)1 mice (*n* = 5). The top row of (G): liver tissue; red arrows indicate obscure nucleus, yellow arrows indicate vacuolar degeneration and blue arrows indicate infiltration of macrophages. The intermediate row of (G): lung tissue; red arrows indicate foci of hemorrhage, yellow arrows indicate edema of lung tissue and blue arrows indicate infiltration of macrophages. The bottom row of (G): kidney tissue; red arrows indicate cell lysis and necrosis and blue arrows indicate infiltration of inflammatory cells. Scale bar = 25 μm. Magnification, 400 × . The number of the scored fields = 15. The results were presented as mean ± SD through three independent experiments. The results of (A) are analyzed by Kaplan–Meier survival curves and Log‐rank test (Mantel–Cox method) and the results of (B–H) were analyzed by one‐way ANOVA. *p < 0.05, ** *p *< 0.01, *** *p *< 0.001, ****p < 0.0001. ns, no significance

To investigate the therapeutic effectiveness of mIL‐6R mAbs for SIRS, we established the murine SIRS model by intraperitoneal injection of LPS. We utilized the commercialized IL‐6R mAb Tocilizumab, of which SIRS is a candidate disease, as treatment for the positive control group and analyzed the survival time of LPS‐induced SIRS mice with the h‐mIL‐6R or Tocilizumab treatment.^8^ The results showed that both the h‐mIL‐6R and Tocilizumab significantly improved the survival of the SIRS mice (Figure 3A), indicating the effectiveness of mIL‐6R blockade in the treatment of SIRS. Besides, to evaluate the capability in controlling systemic inflammation response mediated by the mIL‐6R mAb treatment, we measured the levels of pro‐inflammatory cytokines in serum including TNF‐α, IL‐1β, IL‐6, and interferon‐γ (IFN‐γ) of the indicated groups 6 h after the treatments, because increased levels of pro‐inflammatory cytokines in serum is the prominent characteristic in the SIRS/sepsis model. The results showed that the h‐mIL‐6R mAb and Tocilizumab treatment significantly decreased serum concentrations of these four cytokines in the LPS‐induced SIRS mice, implying that the IL‐6R mAb treatment could contribute to alleviating systemic inflammation in SIRS (Figure 3B). In view of LPS‐induced inflammatory injury and liver dysfunction, we detected liver injury biomarkers aspartate transaminase (AST) and alanine transaminase (ALT) in serum 24 h after the treatments. The results showed that the IL‐6R mAbs remarkably decreased ALT and AST levels in the serum (Figure 3C). Furthermore, to determine the inflammation of the lung tissue, we detected levels of myeloperoxidase (MPO), the sensitive biomarker associated with neutrophil accumulation‐mediated tissue injury in lung tissues.^28^, ^29^ The results suggested that MPO levels were significantly lowered in the h‐mIL‐6R mAb‐ and Tocilizumab‐treated SIRS mice (Figure 3D), suggesting that inflammation in lung tissue was lessened by the IL‐6R mAbs. Given that kidney injury is frequently complicated with SIRS, we detected blood urea nitrogen (BUN) and creatinine levels to evaluate the alterations of the renal function in the murine SIRS model. We found that both of the mIL‐6R blockade treatments prominently reduced the levels of BUN and creatinine (Figure 3E,F) and they could mitigate renal dysfunction in LPS‐induced SIRS.

In addition, histological malformation can be observed and be used for evaluating severity of organ dysfunctions by scoring analysis in SIRS.^30^ As for liver injury, disappearance of sinus hepaticus, swollen hepatocytes, and slight infiltration of periportal neutrophils and macrophage were observed in lower degree of liver injury, while vacuolar degeneration, ballooning degeneration, and obscure nucleus of hepatocytes and severe inflammatory cell infiltration indicate moderate liver injury. The above alterations together with granulomas and necrosis of hepatocytes are found in higher degree of liver injury. H&E staining showed that the alterations including obscure nucleus (indicated by red arrows) and vacuolar degeneration (indicated by yellow arrows) in hepatocytes of the LPS‐induced SIRS model are apparent (Figure 3G,H). The h‐mIL‐6R mAb decreased these malformations and infiltration of macrophages (indicated by blue arrows) in liver tissue of the SIRS mice. As for lung injury in SIRS, alveolar edema and congestion, macrophage infiltration, and small area of hemorrhage suggest lower degree of lung injury. Consolidation of lung tissue, large area of hemorrhage, and severe macrophage infiltration indicate moderate lung injury, and more hemorrhage, together with severe consolidation of lung tissue implies higher degree of lung injury in SIRS.^30^ The results illustrated that while multiple foci of hemorrhage (indicated by red arrows), edema of lung tissue (indicated by yellow arrows) and severe macrophage infiltration (indicated by blue arrows) were found in the SIRS mice without therapy, both IL‐6R mAbs alleviated histological injuries and reduced scores of lung tissue damage (Figure 3G,H). When it comes to kidney injury, histological malformations include cell lysis and necrosis, loss of brush border, tubular dilation, and inflammatory cell infiltration.^31^ We found that the h‐mIL‐6R and Tocilizumab significantly attenuated cell lysis and necrosis (indicated by red arrows) and infiltration of inflammatory cells (indicated by blue arrows, Figure 3G,H). These results indicated that the h‐mIL‐6R mAb and Tocilizumab relieved inflammatory responses and improved prognosis together with organ injury in the murine SIRS model.

### 2.4 The h‐mIL‐6R mAb mediated anti‐inflammatory effects via the inhibition of NF‐κB/Ccl2 signaling pathways in the LPS‐treated THP‐1 cells

**FIGURE 4:**
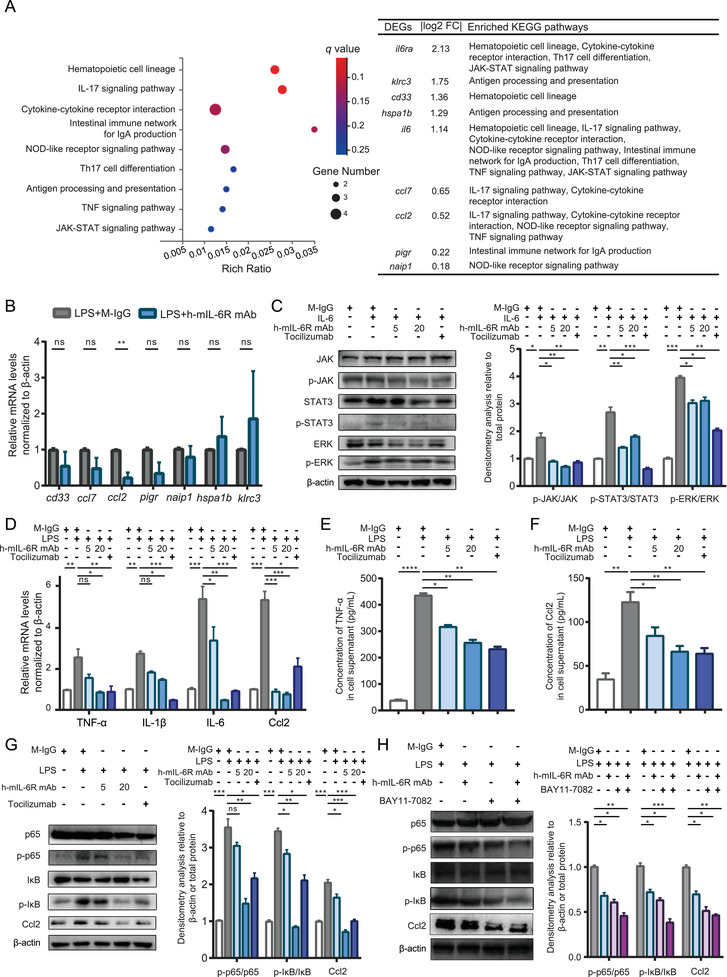
Identification of IL‐6 signaling blockade function and novel mechanism exploration by RNA‐seq for the h‐mIL‐6R mAb‐treated SIRS model in the IL6r‐e(hIL6R)1 mice. (A) The IL6r‐e(hIL6R)1 mice with LPS‐induced SIRS (40 mg/kg, i.p.) were treated with the h‐mIL‐6R mAb (2.5 mg/kg) or M‐IgG (2.5 mg/kg). The PBMC for RNA‐seq analysis was collected 12 h after the treatments (*n* = 3). Left: the bubble diagram displayed the top differential KEGG pathways enriched by DEGs with *q* value, rich ratio (the proportion of the DEGs to all genes of the enriched pathway) and gene number of the included DEGs. Right: the list of the DEGs (*p* < 0.05, *q* < 0.1) enriched in the pathways showed the absolute value of log2(fold change (FC)) and the corresponding KEGG pathways. (B) The PBMC of the IL6r‐e(hIL6R)1 mice (*n* = 3) with LPS‐induced SIRS was collected for verification of the DEGs included in the enriched KEGG pathways (except *il6* and *il6ra*) by qRT‐PCR. (C) Immunoblotting and densitometry analysis of JAK/STAT3 and JAK/ERK signaling pathways in the co‐treated THP‐1 cells. The THP‐1 cells were treated with the h‐mIL‐6R mAb/Tocilizumab/M‐IgG for 30 min, then added with IL‐6 and co‐treated with the mAbs/M‐IgG and IL‐6 for another 6 h. The eventual concentrations of all the agents were: h‐mIL‐6R mAb with 5 or 20 μg/ml, Tocilizumab with 5 μg/ml, and IL‐6 with 12.5 ng/ml. (D) qRT‐PCR analysis of TNF‐α, IL‐1β, IL‐6, and Ccl2 mRNA levels in the co‐treated THP‐1 cells. The THP‐1 cells were treated with the h‐mIL‐6R mAb/Tocilizumab/M‐IgG for 30 min, then added with LPS and co‐treated with the mAbs/M‐IgG and LPS for 6 h. The eventual concentrations of all the agents were: h‐mIL‐6R mAb with 5 or 20 μg/ml, Tocilizumab with 5 μg/ml, and LPS with 1 μg/ml. (E,F) ELISA and statistical analysis of TNF‐α and Ccl2 levels in the supernatant of the THP‐1 cells with the indicated treatments identical to (D). (G) Immunoblotting and densitometry analysis of p65, IκB, and Ccl2 of the THP‐1 cells with the indicated treatments identical to (D). (H) Immunoblotting and densitometry analysis of p65, IκB, and Ccl2 of the THP‐1 cells with the h‐mIL‐6R mAb (10 μg/mL) and the NF‐κB inhibitor BAY 11–7082 (5 μM) treatment for 30 min in advance followed by co‐treatment of LPS (1 μg/ml) for 6 h. The results were presented as mean ± SD through three independent experiments. The qRT‐PCR and immunoblotting results were normalized to β‐actin and the levels of the phosphorylation proteins were normalized to the corresponding total proteins. The results of (B–H) were analyzed by one‐way ANOVA. *p < 0.05, ** *p *< 0.01, *** *p *< 0.001, ns, no significance

Given that PBMC mediates inflammatory response via recognition and release of cytokines including IL‐6 in SIRS, we focused on the effects of mIL‐6R blockade on inflammatory response in PBMC isolated from LPS‐treated IL6r‐e(hIL6R)1 mice.^32^ To explore the mechanism underlying the inhibitory effect of mIL‐6R blockade mediated by the h‐mIL6R mAb in the LPS‐induced murine SIRS model, we collected PBMC from the model with/without the h‐mIL6R mAb (25 mg/kg) treatment and employed RNA‐seq analysis to find out the differential expressed gene (DEG) profiles. We obtained 65 major DEGs (*p* < 0.05, *q* < 0.1) and then enriched the 65 DEGs into KEGG database. Considering the potential effects associated with inflammatory response and immune system caused by mIL‐6R blockade, we selected the first nine significant KEGG pathways (Figure 4A) and the corresponding DEGs enriched to them. We found that “JAK‐STAT signaling pathway” and “TNF signaling pathway” associated with IL‐6 signaling were enriched, implying the effectiveness of mIL‐6R blockade mediated by the h‐mIL6R mAb to some extent. What's more, we found that “NOD‐like receptor signaling pathway” concerned with inflammasome and pyroptosis downstream NF‐κB signaling was also included in the enriched pathways, suggesting that mIL‐6R blockade mediated by the h‐mIL‐6R mAb could be involved in NF‐κB/NLRP3 signaling and pyroptosis in PBMC. Next, we got down to verifying expression alteration of the DEGs in the enriched pathways by qRT‐PCR in PBMC. Considering IL‐6R expression and IL‐6/IL‐6R were manipulated in the LPS‐induced murine SIRS model with h‐mIL‐6R mAb treatment, we excluded these two genes and verified the rest seven DEGs. The results showed that compared with the LPS+M‐IgG group, Ccl2 was significantly decreased by the h‐mIL‐6R mAb treatment in the LPS‐induced SIRS model (Figure 4B). By contrast, other examined DEGs showed no significance with/without the h‐mIL‐6R mAb treatment. Previous studies reported that Ccl2 magnified the recruiting effect of inflammatory cells and expression of pro‐inflammatory mediators of monocyte/macrophage.^33^, ^34^ What's more, Ccl2 expression was able to be activated by IL‐6 treatment and by the transcription factor p65 of NF‐κB signaling pathway with LPS treatment.^21^, ^35^ Therefore, mIL‐6R blockade mediated by the h‐mIL‐6R mAb may protect from inflammatory responses and SIRS by regulating Ccl2 expression.

Forwardly, for verification of RNA‐seq results and mechanism investigations in vitro, we used the human monocyte cell line THP‐1, highly expressing IL‐6R, for simulation of PBMC according to previous studies, because monocyte/macrophage is the important component of PBMC and exerts the role of effector cell of the innate immune system responding to LPS‐induced systemic inflammatory response.^36^, ^37^ To verify the effectiveness of the h‐mIL‐6R mAb in mIL‐6R blockade at first, we detected activation of JAK/STAT3 and JAK/MAPK (extracellular signal‐regulated kinase, ERK) signaling pathway with IL‐6 treatment, because IL‐6/mIL‐6R complex leads to activation of JAK and subsequent phosphorylation of cytoplasmic STAT3 and MAPK via gp130. We treated THP‐1 cells with the h‐mIL‐6R mAb/Tocilizumab/M‐IgG for 30 min, then added IL‐6 to the cells and cultured the THP‐1 (co‐treated with the mAbs/M‐IgG and IL‐6) for another 6 h. Immunoblotting analysis showed that while IL‐6 treatment significantly increased phosphorylation levels of JAK, STAT3 and ERK, the h‐mIL‐6R mAb inhibited phosphorylation of JAK, STAT3 and ERK, indicating that the h‐mIL‐6R mAb effectively blocked mIL‐6R and suppressed activation of JAK/STAT3 and JAK/MAPK signaling pathway induced by IL‐6 treatment (Figure 4C). These results demonstrated that the h‐mIL‐6R mAb was capable of blocking IL‐6/mIL‐6R signaling and inhibiting LPS‐induced inflammatory signaling activation, which was in consistent with our results of the LPS‐induced murine SIRS model.

To examine the protective effects from LPS‐induced inflammatory signaling activation and potential effects on Ccl2 expression indicated by RNA‐seq, we treated THP‐1 cells with the h‐mIL‐6R mAb/Tocilizumab/M‐IgG for 30 min, then added LPS to the cell and cultured the THP‐1 (co‐treated with the mAbs/M‐IgG and LPS) for another 6 h. Subsequently, we detected the levels of pro‐inflammatory cytokines including TNF‐α, IL‐1β, IL‐6 and Ccl2 by qRT‐PCR. We found that LPS stimulation promoted TNF‐α, IL‐1β, IL‐6, and Ccl2 gene expression and more importantly, the h‐mIL‐6R mAb decreased mRNA levels of the above pro‐inflammatory cytokines and Ccl2 (Figure 4D). More importantly, we detected levels of TNF‐α and Ccl2 in the supernatant of the THP‐1 by ELISA because they exert the pro‐inflammatory effects following the secretion from the cells. We found that the h‐mIL‐6R and Tocilizumab deceased TNF‐α and Ccl2 levels in the supernatant, suggesting that the mIL‐6R blockade inhibited secretion of TNF‐α and Ccl2 from the LPS‐treated THP‐1 cells (Figure 4E,F). Considering activation of NF‐κB is critically required for LPS‐induced inflammation, we studied the effects of the h‐mIL‐6R mAb on the regulation of the NF‐κB signaling pathway. We detected phosphorylation of the key subunit of NF‐κB, p65 and the inhibitive regulation factor inhibitor of NF‐κB (IκB) by immunoblotting analysis (Figure 4G). We found that the h‐mIL‐6R mAb and Tocilizumab could promote phosphorylation of IκB and p65, suggesting that both of the mIL‐6R blockade mAbs effectively inhibited LPS‐induced activation of the NF‐κB signaling pathway. To verify whether mIL‐6R blockade could regulate Ccl2 expression, we also detected Ccl2 levels and found that the h‐mIL‐6R mAb remarkably inhibited LPS‐induced Ccl2 expression. To clarify whether NF‐κB regulates mIL‐6R blockade‐mediated Ccl2 inhibition, we utilized NF‐κB inhibitor BAY 11‐7082 in addition to the h‐mIL‐6R mAb and LPS co‐treatment. The results showed that the combination of the NF‐κB inhibitor and the h‐mIL‐6R mAb forwardly decreased LPS‐induced Ccl2 expression in the THP‐1 cells (Figure 4H). Collectively, mIL‐6R blockade mediated by the h‐mIL‐6R mAb or Tocilizumab was able to inhibit the pro‐inflammatory signaling NF‐κB/Ccl2 in LPS‐treated monocyte.

### 2.5 The h‐mIL‐6R mAb inhibited NLRP3‐mediated inflammasome formation and pyroptosis in the LPS‐treated THP‐1 cells

**FIGURE 5:**
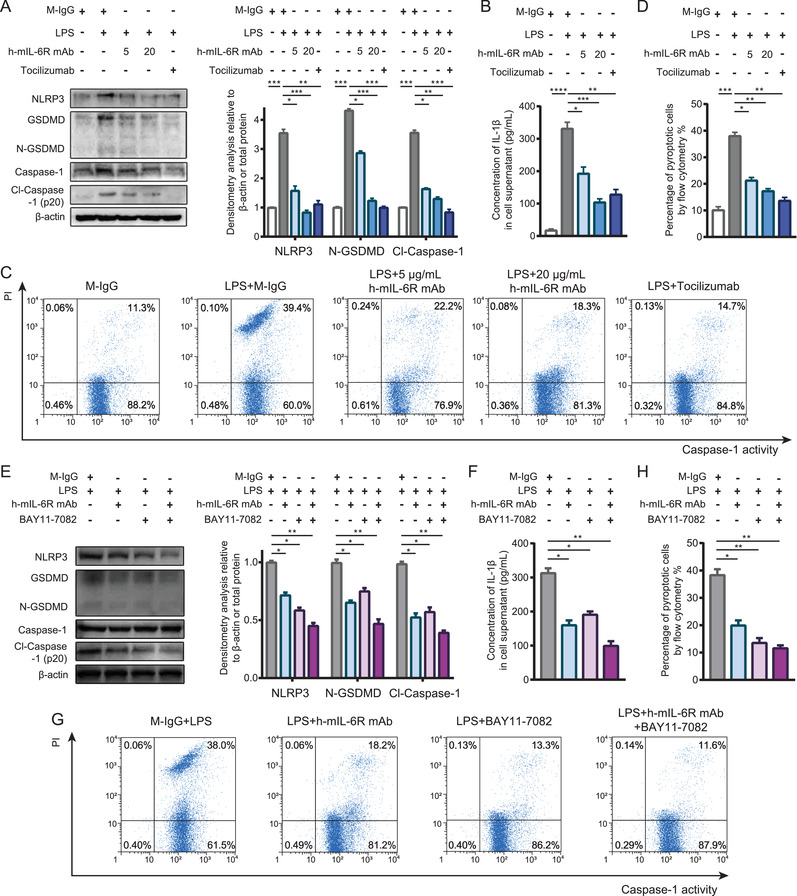
The IL‐6R mAbs alleviated pyroptosis of THP‐1 by regulating NLRP3‐mediated inflammasome formation. (A) Immunoblotting and densitometry analysis of NLRP3, GSDMD, Caspase‐1 in the THP‐1 cells treated with the h‐mIL‐6R mAb (5 and 20 μg/ml) or Tocilizumab (5 μg/ml) for 30 min in advance, followed by treating with LPS (1 μg/ml) for 6 h. (B) ELISA and statistical analysis of IL‐1β levels in the supernatant of the THP‐1 cells with the indicated treatments identical to (A). (C,D) Flow cytometry analysis of cell pyroptosis with the indicated treatments in the THP‐1 cells. (E) Immunoblotting and densitometry analysis of NLRP3, GSDMD, Caspase‐1 in the THP‐1 cells treated with the h‐mIL‐6R mAb (10 μg/ml) or the NF‐κB inhibitor BAY 11–7082 (5 μM) for 30 min in advance, followed by treating with LPS (1 μg/ml) for 6 h. (F) ELISA and statistical analysis of IL‐1β levels in the supernatant of the THP‐1 cells with the indicated treatments identical to (E). (G,H) Flow cytometry analysis of cell pyroptosis with the indicated treatments in the THP‐1 cells. Cl‐Caspase‐1, cleaved Caspase‐1. The results were presented as mean ± SD through three independent experiments. The protein levels of N‐GSDMD and cleaved Caspase‐1 were normalized to the corresponding total proteins. The results were analyzed by one‐way ANOVA. *p < 0.05, ** *p *< 0.01, *** *p *< 0.001, ns, no significance

Although the KEGG enrichment analysis of the RNA‐seq results indicated that IL‐6R blockade mediated by the h‐mIL‐6R mAb could regulate “NOD‐like receptor signaling pathway” and the expression of the enriched DEG Naip1, it has been documented that Naip1 is the receptor of the bacterial protein, type III secretion system (T3SS) needle protein, and activates NLRC4‐mediated inflammasome.^27^ To our knowledge, LPS‐induced SIRS model did not bring about bacterial protein exposure and NLRC4 activation. More importantly, we found that the indicated DEG Naip1 was not significantly regulated as demonstrated by the qRT‐PCR results in the PBMC isolated from the LPS‐induced murine SIRS model. However, NF‐κB was found to promote pro‐inflammatory mediator expression including TNFs and IFNs, but also NLRP3 priming, the ready state of inflammasome activation.^27^ Besides, it has been documented that NLRP3 inflammasome is critical in LPS‐induced sepsis and septic organ injury.^38^ Considering the inhibition of IL‐6R blockade mediated by the h‐mIL‐6R mAb on pro‐inflammatory NF‐κB signaling, we speculated that IL‐6R blockade could also inhibit NLRP3‐associated inflammasome and pyroptosis via inhibiting NF‐κB signaling in the LPS‐stimulated monocyte. We detected levels of NLRP3, the active form of GSDMD (N‐terminal of GSDMD, N‐GSDMD) and Caspase‐1 (p20) by immunoblotting analysis. We found that LPS treatment significantly activated NLRP3 inflammasome, GSDMD and Caspase‐1, whereas the h‐mIL‐6R mAb (5 and 20 μg/ml) prominently decreased the levels of NLRP3, N‐GSDMD and cleaved Caspase‐1 (p20) (Figure 5A). Besides, we performed ELISA to assay the levels of IL‐1β in the supernatant of the treated THP‐1 cells since pyroptotic cell is also characterized by the release of mature IL‐1β through the membrane pores perforated by N‐GSDMD. The results showed that both the h‐mIL‐6R and Tocilizumab significantly decreased IL‐1β levels in the supernatant (Figure 5B). Furthermore, we employed flow cytometry analysis to determine pyroptotic cell (PI ^high^ Caspase‐1 activity^high^) percentage and found that the IL‐6R mAbs reduced the percentage of PI ^high^ Caspase‐1 activity^high^ cells (Figure 5C,D), consistently indicating that the mIL‐6R blockade could prevent THP‐1 cell from pyroptosis. To investigate whether the mIL‐6R blockade alleviated NLRP3‐mediated pyroptosis through inhibiting NF‐κB activation, we utilized the NF‐κB inhibitor BAY 11–7082 and performed immunoblotting analysis of the pyroptosis‐associated proteins including NLRP3, GSDMD, and Caspase‐1. We found that combination of h‐mIL‐6R mAb and the NF‐κB inhibitor led to significant reduced levels of NLRP3, N‐GSDMD, and cleaved Caspase‐1 in THP‐1 cells (Figure 5E). ELISA assay showed that the h‐mIL‐6R mAb and the NF‐κB inhibitor further suppressed release of IL‐1β in the LPS‐treated THP‐1 cells (Figure 5F). Accordingly, the co‐treatment of the h‐mIL‐6R mAb and the NF‐κB inhibitor decreased pyroptotic cell (PI ^high^ Caspase‐1 activity^high^) percentage (Figure 5G,H). These results suggested the mIL‐6R blockade could suppress inflammatory signaling by regulating NLRP3‐associated pyroptosis via NF‐κB inhibition in monocyte.

## 3 DISCUSSION

In this study, we generated the human *il‐6r* gene knock‐in mice IL6r‐e(hIL6R)1 and prepared a defined epitope murine mAb against human mIL‐6R (h‐mIL‐6R mAb) with high affinity. In the LPS‐induced murine SIRS model, we found that both the commercial IL‐6R mAb Tocilizumab and the prepared h‐mIL‐6R mAb alleviated systemic inflammatory responses, organ dysfunctions, and improved survival rates. To explore the underlying mechanisms of mIL‐6R blockade in SIRS treatment, we employed RNA‐seq and found that expression of the pro‐inflammatory chemokine Ccl2 significantly decreased after the mIL‐6R mAb treatment. *I* *n vitro* studies indicated that the mIL‐6R blockade not only inhibited IL‐6/JAK/STAT3 and IL‐6/JAK/MAPK pathways but also decreased Ccl2 expression via suppressing NF‐κB signaling pathway in the monocyte cell line THP‐1. Besides, we found that the mIL‐6R blockade mitigated pyroptosis through suppressing NF‐κB‐mediated NLRP3 inflammasome, suggested by decreased IL‐1β secretion, cleaved Caspase‐1 and N‐GSDMD levels in the LPS‐treated THP‐1 cells. Taken together, mIL‐6R blockade by the h‐mIL‐6R mAb alleviated excessive inflammatory responses and organ dysfunctions of the LPS‐induced SIRS in the IL6r‐e(hIL6R)1 mice, potentially through inhibiting NF‐κB‐regulated inflammation and pyroptosis of monocyte.

As previously reported, IL‐6 is correlated with SIRS state as well as adverse event incidence, and is regarded as a useful laboratory parameter for objective and quantitative measurement of inflammatory status, indicating the crucial role of IL‐6 in detection, progression, and the prognosis of SIRS.^1^, ^39^ Hence, modulating IL‐6/mIL‐6R signaling is important for preventing progression and improving prognosis of SIRS, and it could be a promising strategy for management of SIRS. Nevertheless, currently approved therapeutic antibodies targeting IL‐6 (such as Sirukumab) and IL‐6R (such as Tocilizumab) are not conventional administration for SIRS. Moreover, Tocilizumab is merely a candidate for SIRS treatment while other IL‐6R antibodies are not approved or clinically trialed for SIRS. Of note, IL‐6R blockade is approved for treatment of CAR‐T‐complicated CRS.^8^ Therefore, IL‐6R blockade could also be the alternative therapy in SIRS patients. This study provided a new insight that mIL‐6R‐targeted therapy was of great significance in the management of SIRS both in vivo and in vitro. Our preliminary results demonstrated that blockade of mIL‐6R‐mediated IL‐6 signaling was the effective strategy for inhibiting inflammatory responses and improving the prognosis of SIRS. Besides, the indication of mIL‐6R‐targeted therapy could be extended to SIRS. In addition to decreasing pro‐inflammatory cytokines, the h‐mIL‐6R mAb also alleviated organ injury complicated to SIRS. Excessive inflammatory response is frequently connected with cell dysfunction and death. Besides, it has been reported that excessive release of IL‐6 is the major cause of multiple organ dysfunction syndrome (MODS) and septic shock.^40^ We found that the tissue damage in LPS‐induced SIRS of liver, lung, and kidney was alleviated by the mIL‐6R mAbs, reflected by histological alterations and scoring analysis. Furthermore, we examined the indexes of cellular damage and dysfunction including transaminase, MPO, BUN and creatinine, all of which were found to decrease after the treatment of the mIL‐6R mAbs. Therefore, mIL‐6R blockade might be beneficial for organ dysfunctions of SIRS.

Acute liver failure (ALF) in SIRS is a kind of gastro‐hepato‐intestinal emergency that can develop into coma or death.^41^ About 60% of the ALF patients complicates with SIRS.^42^ Increased IL‐6 in serum has been found in LPS‐induced ALF and IL‐6 has been reported to induce liver injury by several mechanisms such as NF‐κB and miR‐455‐3p/PI3K signaling pathways.^43^, ^44^ It has been documented that in hepatocytes, IL‐6 is one of the most important triggers of the acute phase protein expression which contributes to the control of inflammation at systemic levels.^45^, ^46^ Of note, monocytes/macrophages are crucial determinants for acute liver injury and consequent uncontrolled immune activation brings about sepsis as well as organ failure.^47^ A recent study revealed that rapid and drastic elevation of serum IL‐6 was derived from circulating monocytes and ablation of monocyte‐derived IL‐6 in vivo alleviated liver dysfunction and systemic inflammation.^48^ In consistence with this study, we focused on monocyte and found that the IL‐6R mAbs suppressed the inflammatory signaling pathways and decreased the production of inflammatory mediators. Further studies concerning the interaction of circulating monocyte/macrophage and hepatocyte are of necessity to reveal the detailed effects of IL‐6/mIL‐6R signaling blockade in monocyte on regulating homeostasis of hepatocytes in SIRS/sepsis.

Acute lung injury (ALI) is a syndrome characterized by damage to the alveolar‐capillary wall, pulmonary edema, and recruitment of inflammatory cells with mortality up to 40%–60%.^28^, ^49^ It is reviewed that IL‐6, TNF‐α, and IFN‐γ are among the clinical predictors and biomarkers of adverse outcomes in ALI and acute respiratory distress syndrome (ARDS).^50^ Inhibition of IL‐6/STAT3 signaling suppressed macrophage activation, T helper 17 cell differentiation, and attenuated inflammatory responses, thereby bringing benefits to lung injury and ARDS.^51^ The h‐mIL‐6R mAb attenuated lung tissue damage demonstrated by the results of histological damage scoring and MPO assay, indicating that the IL‐6R blockade was beneficial to lung tissue damage. In fact, several lines of evidence suggested that inhibition of IL‐6/STAT3 pathway exerts protective effects against LPS‐induced ALI, implying that IL‐6R‐targeted therapy is an effective strategy to alleviate ALI in SIRS.^52^, ^53^

Acute kidney injury (AKI) in SIRS/sepsis is closely associated with patient outcomes.^54^ While the biomarkers for early diagnosis or predicting prognosis of SIRS‐related renal dysfunction are limited in availability and feasibility, the elevation of BUN and creatinine are common and persuasive for indicating AKI in SIRS.^55^ Our results showed that IL‐6R blockade prominently decreased both BUN and creatinine levels, and mitigated histological alterations in kidney tissue from LPS‐induced SIRS mice, indicating the improvement of renal dysfunction in SIRS. It has been stated that AKI could exacerbate dysfunction of other organs due to impaired elimination of toxins and metabolites.^56^ More importantly, IL‐6 signaling activation is involved in organ dysfunctions of liver, lung, intestine, and cardiorenal syndrome complicated to AKI.^57^, ^58^ The present study consistently demonstrated that liver, lung, and kidney injuries were accompanied with IL‐6 increasing, and the mIL‐6R mAbs‐alleviated multiple organ dysfunctions, suggesting that prevention of IL‐6 signaling overactivation could reduce systemic inflammation and mitigate tissue injuries in SIRS.

In this study, we employed traditional hybridoma technology to generate the epitope‐defined mIL‐6R mAb. In order to make an epitope‐defined human IL‐6R mAb without reaction to mouse IL‐6R, we used different peptides from the antigen but not the full‐length protein to immunize and screen the specific antibody against human IL‐6R. To select a right and suitable peptide antigen for antibody development, the following multiple aspects should be considered, including antigenicity, hydrophilicity, hydrophobicity, surface probability, transmembrane, homology, flexible region, helix region, sheet region, signal peptide, and modification. In the current study, we used two software, Antheprot 4.3 and OptimumAntigen™ design tool in order to conduct in‐depth analysis of the human IL‐6R (NP_000556.1) sequence‐related characteristics. We selected the candidate peptides as the possible epitope by analyzing accessibility on cell surface, local hydrophilicity, the existence of post‐translational modifications, etc. The following four peptides are selected and finally used as the possible candidate immunogens to prepare antibody: Pep1 (441‐455aa): NTSSHNRPDARDPRS, Pep2 (315‐329aa): WTESRSPPAENEVST, Pep3 (133‐146aa): EWGPRSTPSLTTKA; Pep4 (393‐406aa): KLRALKEGKTSMHP. We determined Pep3 as the final antigen and obtained the specific mAb clone after several rounds of selection and identification according to the conventional hybridoma technology protocols.

It has been documented that two major downstream signaling pathways activated by IL‐6/IL‐6R are JAK/STAT3 and JAK/MAPK. These pathways promote acute phase protein expression and activities of transcription factors such as activator protein‐1 (AP‐1) family, thereby modulating inflammatory response and process.^59^ Besides, the NF‐κB signaling pathway has long been considered as the classical pathway which modulates the activation of inflammation after infection. Once activated, NF‐κB translocates into the nucleus to regulate the target genes including pro‐inflammatory cytokines and chemokines. Therefore, suppression of JAK and NF‐κB signaling pathways essentially regulated the initiation and progression of systemic inflammation. Our results illustrated that IL‐6R blockade suppressed JAK/STAT3, MAPK, and NF‐κB signaling pathways. RNA‐seq and qRT‐PCR implied that Ccl2 levels significantly reduced with the h‐mIL‐6R mAb pretreatment in PBMC of LPS‐induced SIRS mice. Ccl2 is responsible for monocyte recruitment in the inflammatory conditions. Suzuki et al. have reported that IL‐6 was able to induce Ccl2 production in PBMC while IL‐6 and sIL‐6R increased IL‐6 production in synovial cell and human umbilical vessel endothelial cell (HUVEC). Of note, IL‐6 was unveiled to promote the adhesion of monocyte to HUVEC, suggesting that IL‐6 was the positive regulator of chemokine expression but also the chemotaxis of monocyte.^60^ Besides, the transcription factor NF‐κB is capable of activating Ccl2 expression by binding to the distal enhanced region near to the 5′‐terminus of Ccl2.^35^ Our in vivo studies indicated that the mIL‐6R blockade decreased Ccl2 expression in PBMC of SIRS mice. We forwardly demonstrated that the combination of mIL‐6R blockade and NF‐κB inhibition significantly decreased Ccl2 expression compared to sole suppression of either mIL‐6R or NF‐κB in vitro. Therefore, IL‐6/mIL‐6R signaling could potentially regulate Ccl2 expression by suppression of NF‐κB.

NLRP3 is the sensor and integration point of cellular stress caused by infection, dysregulated metabolism, extracellular ATP, etc.^61^ It in particular responds to innate immune signaling thereby responsible for assembly of inflammasome. NLRP3 inflammasome mediates the pro‐inflammatory responses and pyroptosis through activation of GSDMD and Caspase‐1. Consequently, NLRP3 inflammasome induces cell death and release of pro‐inflammatory mediators such as IL‐1β and prostaglandins.^62^ It has been revealed that NLRP3‐mediated pyroptosis is capable of exacerbating inflammation in SIRS/sepsis. The previous study found that inflammasome pathway was activated in PBMC of SIRS/sepsis patients.^24^ In the LPS‐induced SIRS model, overexpressed NLRP3 accumulated in circulating monocyte‐derived exosomes could promote maturation of pro‐IL‐1β in the local macrophage and result in the aggravation of inflammation.^63^ More importantly, activation of NF‐κB promotes transcription and expression of NLRP3, indicating that suppressing NF‐κB signaling pathway could reduce NLRP3 level and NLRP3 inflammasome‐mediated pyroptosis.^64^ Given that blockade of mIL‐6R by h‐mIL‐6R mAb and Tocilizumab suppressed NF‐κB signaling pathway, our in vitro studies showed that mIL‐6R could also inhibit inflammasome‐related responses. Furthermore, we found that combination of mIL‐6R blockade and NF‐κB inhibition prominently suppressed pyroptosis and inflammatory cascades. These results implied that IL‐6/mIL‐6R signaling could manipulate inflammatory responses via NF‐κB/NLRP3‐induced pyroptosis.

Altogether, both the h‐mIL‐6R mAb and Tocilizumab showed therapeutic effects of SIRS and organ dysfunctions in the human *il‐6r* knock‐in murine SIRS model. The mIL‐6R blockade regulated JAK/STAT3 and JAK/MAPK activation, but also suppressed NF‐κB/Ccl2 and NLRP3/GSDMD signaling pathways. Targeting mIL‐6R in SIRS management could be a promising strategy to prevent overactivation of inflammatory cascades, protect organs from dysfunction, and improve outcomes of SIRS patients.

## 4 MATERIALS AND METHODS

### 4.1 Generation of the mAbs against human mIL‐6R

The in‐house mAb against human mIL‐6R (h‐mIL‐6R mAb) was generated by traditional hybridoma technology. In brief, for immunization, the immune peptide from human mIL‐6R (Peptide3, sequence: EWGPRSTPSLTTKAC, location: 133–146 amino acids) conjugated with KLH or bovine serum albumin (BSA) was prepared by ChinaPeptides Co. Ltd, Shanghai, China. The C57BL/6J mice were immunized with 50 μg Peptide3‐BSA for three times per 20 days. The supernatants of the hybridoma cells (HCs) were determined by ELISA and flow cytometry assay as previously reported.^65^

### 4.2 Generation and identification of the human *il‐6r* gene knock‐in transgenic mice

CRISPR Cas9 technology was employed to knock in human *il‐6r* gene in C57BL/6J mice and was conducted by Shanghai Biomodel Organism Science & Technology Development Co., Ltd. In brief, the knock‐in site of human *il‐6r* gene located between the exon 1 of murine *il‐6r* gene and its upstream non‐coding region: 5′UTR‐…GCCGCTCTGCCGCCCGCCGTCCCGCGTAGAAGG AAGCATGCTGACCGTCGGCTGCACGCTGTTGGTCGCCCTGCTGGCCGCGCCCGCGGTCGCGCTGGTCCTCGGGAGCTGCCGCGCGCTGG…‐3′UTR. Underlined sequence shown above was the site and the sequence from forward to reverse of gRNA. Cas9 mRNA and gRNA were in vitro transcribed and identified by nucleic acid electrophoresis. Homologous recombinant plasmid IL6r‐e(hIL6R)1 Donor Vector containing 5′ homology arm‐*h‐il‐6r* cDNA‐woodchuck hepatitis virus post‐transcriptional regulatory element (wpre)‐polyA‐3′ homology arm was constructed. The restriction enzyme *Xba* I enzyme was used for digestion. The homologous recombinant plasmid was identified by nucleic acid electrophoresis. Then, the plasmid was transfected into *E. coli* followed by ampicillin selection and plasmid DNA extraction. After male and female C57BL/6J mice mated, Cas9 mRNA and gRNA were mixed and injected into the zygotes. The transfected zygotes were cultured until the blastocyst stage, followed by being transferred into pseudopregnant mice. The parental generation (P_0_) transgenic mice were generated and identified at 3 weeks old by PCR to select those with correct homologous recombination. The identification strategy by PCR was to amplify a fragment from 5′ homology arm and 3′ homology arm with a specific length, respectively. The used primers were: 5′ homology arm forward 5′‐CACGTTTCCCCCTTAGCCTCTTCA‐3′, reverse 5′‐ACACTTTACTTCCGGGGGTCTCAT‐3′, 3′ homology arm forward 5′‐CTGCGCGGGACGTCCTTCTGCTAC‐3′, and reverse 5′‐ TCCGGCCCCTGAGTCCTATT‐3′. The PCR was performed using PrimeStar GXL kit (Takara Bio Inc, Tokyo, Japan) and the procedure was: 98°C for 2 min followed by 34 cycles of denaturation at 98°C for 10 s, annealing at 68°C for 15 s, and extension at 68°C for 3 min. The selected P_0_ transgenic mice mated with C57BL/6J mice of WT to generate the first offspring (P_1_) which were identified by PCR. The strategy and procedure were identical to that of P_0_ identification. When P_0_ and P_1_ were identified, they were transferred to the Experimental Animal Center of our department in Air Force Medical University for breeding. The identification strategy for subsequent generations of mice by PCR was to amplify a fragment from 5′ UTR to 3′ UTR of WT allele and targeted allele with specific length, respectively. The primers were: WT allele forward 5′‐GTGGCGCGGCTGCAGGAAGTAACC‐3′, reverse 5′‐CGAACCTCCACCGCGTCAGCACAG‐3′, targeted allele forward 5′‐ACGCTGCTTTAATGCCTTTGTATC‐3′, reverse 5′‐GGGCTCACTCTGCGAACGGAACC‐3′. The procedure was: 94°C for 5 min followed by 35 cycles of denaturation at 94°C for 30 s, annealing at 65°C for 30 s, and extension at 72°C for 1 min.

### 4.3 Animal studies and ethics statement

C57BL/6J mice were purchased from Experimental Animal Center of Air Force Medical University. The mice were kept under well‐controlled environmental conditions with constant temperature (25 ± 2)°C, humidity (60 ± 10)%, and alternating 12 h‐light–dark cycles. Standard pellet diet and sterilized water were accessible ad libitum.

To establish the murine SIRS model, LPS (*E. coli* O111:B4, Sigma Aldrich, St. Louis, MO, USA) was diluted with 0.9% sterilized saline. 8‐week‐old male IL6r‐e(hIL6R)1 mice weighing (20 ± 2) g were chosen and were divided into six groups: 0.2 ml saline (intraperitoneal injection, i.p.) as the negative control group, 0.1 ml LPS and 0.1 ml mouse IgG (1 mg/ml) i.p., 0.1 ml LPS and 2.0 mg Tocilizumab (2 mg/ml, 0.1 ml) i.p. as the positive control group, 0.1 ml saline and 2.5 mg h‐mIL‐6R mAb (25 mg/ml, 0.1 ml) i.p., 0.1 ml saline and 0.5 mg h‐mIL‐6R mAb (5 mg/ml, 0.1 ml) i.p., 0.1 ml saline and 0.1 mg h‐mIL‐6R mAb (1 mg/ml, 0.1 ml) i.p.

The investigation was performed in accordance with the Regulations for the Administration of Affairs Concerning Experimental Animals approved by the State Council of People's Republic of China and was approved by the Ethical Committee for Animal Experimentation of Air Force Medical University.

### 4.4 Cell culture

HepG2, U251MG and THP‐1 cell lines were purchased from ATCC (Manassas, VA, USA). All the cells were authenticated by short‐tandem repeat (STR) DNA testing by the Air Force Medical University Center for DNA Typing. HepG2 and hybridoma cell lines were cultured in DMEM medium (Gibco, NY, USA) supplemented with 10% fetal bovine serum (FBS, Invitrogen, Carlsbad, CA, USA). U251MG and THP‐1 cell lines were cultured in RPMI 1640 (Invitrogen, CA, USA) containing 10% FBS. All the cells were incubated in humidified atmosphere of 5% CO_2_ at 37°C and tested for mycoplasma contamination. The NF‐κB inhibitor BAY 11–7082 was purchased from Selleck Chemicals (S2913, TX, USA). Human IL‐6 was purchased from PeproTech (200‐06‐5UG, NJ, USA).

### 4.5 RNA extraction and qRT‐PCR

Total RNA was extracted using E.Z.N.A. Total RNA Kit II (OMEGA Bio‐tek, Inc, GA, USA) according to the manufacturer's instructions. Reverse transcription was performed using PrimeScript RTase (Takara Bio Inc) according to the manufacturer's protocol. The expression levels of IL‐6R mRNA were determined with qRT‐PCR using Premix Ex Taq (Takara Bio Inc) according to the manufacturer's instructions and normalized to the expression levels of the endogenous control, β‐actin (*Actb*). The cycling conditions were as follows: 95°C for 2 min, followed by 40 cycles of denaturation at 95°C for 5 s, annealing at 55°C for 10 s, and extension at 72°C for 45 s. All reactions were run in triplicate. Threshold cycle values were used to calculate the fold change in the transcript levels by using the 2^−ΔΔ^ *^Ct^* method. The primers used for qRT‐PCR were listed in Table S1.

### 4.6 RNA interference

siRNA specifically targeting human IL‐6R and negative control siRNA (siNC) were synthesized by GenePharm (Shanghai, China). Sequences of the siRNA were: siRNA1, sense 5′‐GGAAGACAAUGCCACUGUUTT‐3′, antisense 5′‐AACAGUGGCAUUGUCUUCCTT‐3′; siRNA2, sense 5′‐GCUCUUGGUGAGGAAGUUUTT‐3′, antisense 5′‐AAACUUCCUCACCAAGAGCTT‐3′; siNC, sense 5′‐UUCUCCGAACG UGUCACGUTT‐3′, antisense 5′‐ACGUGACACGUUCGGAGAATT‐3′. The siRNA was transfected into cells using Lipofectamine™ 2000 (Invitrogen), and the transfection procedures were performed according to the manufacturer's instructions.

### 4.7 Immunoblotting and antibodies

Immunoblotting analysis was performed as previously described. In brief, cells or tissue samples were lysed in RIPA buffer (Beyotime Biotechnology, Jiangsu, China) added with PMSF (Beyotime). Protein concentration was measured using the bicinchoninic acid (BCA) method kit (Solarbio, Beijing, China). Protein samples were separated by 10% SDS‐PAGE (Beyotime) and then transferred to polyvinylidene fluoride (PVDF) membranes (Millipore, MA, USA). After blocking with 5% non‐fat milk for 1 h, the membrane was incubated with primary antibodies at 4°C overnight and with the corresponding horse radish peroxidase (HRP)‐conjugated secondary antibody for 1 h at room temperature. Finally, the blots were detected using enhanced chemiluminescence substrate (ECL kit, Millipore). The primary antibodies used for immunoblotting were against IL‐6R (mouse monoclonal, Santa Cruz Biotechnology, sc‐373708, diluted with 1:500), β‐actin (rabbit monoclonal, abcam, ab179467, diluted with 1:3000), JAK2 (mouse monoclonal, Cell Signaling Technology, #3230, diluted with 1:2000), Phospho‐JAK1 (Tyr1034/1035)/JAK2 (Tyr1007/1008) (mouse monoclonal, Cell Signaling Technology, #66245, diluted with 1:2000), STAT3 (mouse monoclonal, Cell Signaling Technology, #9139, diluted with 1:1000), Phospho‐STAT3 (Tyr705) (rabbit monoclonal, Cell Signaling Technology, #9145, diluted with 1:1000), ERK1/2 (rabbit monoclonal, ProteinTech, 11257‐1‐AP, diluted with 1:1000), Phospho‐p44/42 MAPK (ERK1/2) (Thr202/Tyr204) (rabbit monoclonal, Cell Signaling Technology, #4370, diluted with 1:2000), p65 (rabbit polyclonal, ProteinTech, 10745‐1‐AP, 1:2000), p‐p65 (Ser536) (rabbit monoclonal, Cell Signaling Technology, #3033, 1:1000), IκBα (mouse monoclonal, Cell Signaling Technology, #4814, diluted with 1:1000), Phospho‐IκB (Ser32) (rabbit monoclonal, HUABio, #2859, diluted with 1:1000), Ccl2 (rabbit monoclonal, Cell Signaling Technology, #39091, diluted with 1:1000), NLRP3 (rabbit polyclonal, abcam, ab214185, diluted with 1:1000), GSDMD (rabbit monoclonal, Affinity Biosciences, #AF4013, diluted with 1:1000), cleaved N‐terminal GSDMD (rabbit monoclonal, abcam, ab215203, diluted with 1:500), cleaved Caspase‐1 (rabbit monoclonal, Cell Signaling Technology, #89332, diluted with 1:1000), Caspase‐1 (rabbit monoclonal, Cell Signaling Technology, #3866, diluted with 1:1000) and the second antibodies were HRP‐conjugated antibodies (goat anti‐rabbit IgG, horse anti‐mouse IgG, #7074, #7076, Cell Signaling Technology, diluted with 1:2000).

### 4.8 Flow cytometry analysis

To evaluate the specificity to human mIL‐6R of the mAbs produced by the HCs, the supernatants of the HCs were collected and the total protein concentration was adjusted to 100 μg/ml. The mAbs from the No. 2 and 5 HCs were collected, purified and the total protein mAb was adjusted to 100 and 1 μg/ml, respectively. The serum of the immunized mice was collected and was used as the polyclonal antibody for the control group. The total protein concentration of the serum was adjusted to 100 μg/ml. The mouse IgG (100 μg/ml) was used for the negative control group. The human mIL‐6R expressing cell line HepG2 was harvested, washed twice with 4°C phosphate buffered saline (PBS) and re‐suspended with the above‐mentioned antibodies and supernatants before being incubated at 37°C for 1 h. Then, the samples were washed and incubated with the second antibody (Donkey anti‐Mouse IgG (H+L) Highly Cross‐Adsorbed Secondary Antibody, Alexa Fluor 488 (A‐21202, Invitrogen, CA, USA)) for 30 min before the flow cytometry detection (Beckman Coulter, FL, USA) using the excitation sources and filters appropriate for fluorescein isothiocyanate (FITC).

Cell pyroptosis was detected with FAM FLICA™ Caspase‐1 Kit (ICT097, Bio‐Rad Laboratories Inc., CA, USA) according to the manufacturers’ instruction. In brief, THP‐1 cells were collected, washed twice with 4°C PBS, and re‐suspended in the diluted buffer (1 ×) with the FAM‐YVAD‐FMK FLICA Reagent of the kit. After incubation at 37°C for 1 h, the propidium iodide (PI) of the kit was added to the stained cells 15 min before the flow cytometry detection using the excitation sources and filters appropriate for FITC.

### 4.9 Resonance‐based biosensor assay

SPR‐based biosensor assay was conducted using the Octet Red96 system (ForteBio, Menlo Park, CA, USA). Briefly, the h‐mIL‐6R mAb was biotinylated using the EZ‐Link™ NHS‐LC‐LC‐Biotin kit (Thermo Scientific™) and diluted with the loaded concentration of 20 μg/ml, while the IL‐6R‐KLH protein solution (IL‐6R‐KLH dissolved in PBS) was diluted in the two‐fold serial dilution with the final concentrations ranging from 1538 to 24 nM. The Biosensor/Streptavidin (SA) Tray (ForteBio, 18‐5019) equilibrated in PBS as the probe was transferred into the IL‐6R‐KLH protein solution. Dissociation constants were calculated from raw data by the analysis software of the Octet Red 96 system (version 6.3, ForteBio).

### 4.10 Detection of cytokines and transaminases by ELISA

ELISA kits for the cytokines TNF‐α, IL‐1β, IL‐6, IFN‐γ, and Ccl2 were purchased from Dakewe Bio‐engineering Co, Ltd (Guangdong, China. Cat. No: 1217202, 1117202 for TNF‐α, 1210122, 1110122 for IL‐1β, 1210602 for IL‐6, 1210002 for IFN‐γ and 1117392 for Ccl2). The procedure was performed according to the manufacturers’ instructions. The plates were measured by using an ELx 808 ELISA reader (Bio‐Tek Instruments Inc., Winooski, VT, USA) at 450 nm. Detection of AST and ALT in the serum was conducted using the pre‐coated ELISA kits (Cloud‐Clone Corp., Hubei, China, Cat. No: SEA207Mu for ALT, SEB214Mu for AST). Blood samples were collected 6 h after LPS i.p. for detection of TNF‐α, IL‐1β, IL‐6, and IFN‐γ, and 24 h after LPS i.p. for AST and ALT detection. Following the collection, the samples were centrifuged at 400 *g* for 20 min at 4°C, and the supernatant was collected for ELISA detection.

### 4.11 Detection of MPO, blood urea nitrogen and creatinine

The lung tissue was collected 12 h after LPS i.p. for MPO detection to evaluate inflammation of lung tissue using the commercial kit (Jiancheng Bioengineering Institute, Jiangsu, China, Cat. No: A044‐1‐1). The mice were sacrificed and 100 mg lung tissue was sectioned, removed to PBS (Shanghai Double‐helix Biotech Co. Ltd) and grinded to be homogenized on ice. The homogenization was centrifuged (15000 *g* for 15 min at 4°C) and the supernatant was collected and stored at −80°C before MPO detection. All the procedures were done according to the manufacturers’ instructions. The plates were measured with an ELx 808 ELISA reader at 460 nm.

BUN and creatinine in serum were detected to evaluate renal dysfunction. Blood samples were collected 12 h after LPS i.p., centrifuged at 400 *g* for 20 min at 4°C, and the supernatant was collected to detect BUN and creatinine levels using commercial kits (Jiancheng, Cat. No: C013‐2‐1 for BUN and C011‐2‐1 for creatinine). All the procedures were done according to the manufacturers’ instructions. The plates were measured with an ELx 808 plate reader (Bio‐Tek Instruments Inc.) at 640 nm for BUN and 546 nm for creatinine.

### 4.12 Hematoxylin and **Eosin** (H&E) **Stainin**g

Tissues of C57BL/6J mice were harvested 24 h after LPS i.p., immersed in 4% paraformaldehyde (pH 7.4) fixative at 4°C overnight and then embedded in paraffin. Fixed sections (5‐μm thick) were cut and stained with hematoxylin‐eosin reagents (Sigma–Aldrich) and finally observed with a light microscopy (Olympus, Tokyo, Japan). For statistical analysis, three fields (magnification, 200 ×) were observed from one slide for scoring. The histopathological scores of liver, lung, and renal injury were calculated as previously reported.^30^, ^31^

### 4.13 RNA‐seq and analysis

Total RNA was extracted from PBMC of transgenic mice using Trizol (Invitrogen) according to manual instruction. Total RNA was qualified and quantified using the Nano Drop and Agilent 2100 bioanalyzer (Thermo Fisher Scientific, MA, USA). RNA‐seq analysis was performed by BGI Group (Guangdong, China). The sequencing data were filtered with SOAPnuke (Ver. 1.5.2). The clean reads were mapped to the reference genome by using HISAT2 (Ver. 2.0.4). Bowtie2 (Ver. 2.2.5) was applied to align the clean reads to the reference coding gene set, and then the expression level of gene was calculated by RSEM (Ver. 1.2.12). Differential expression analysis and KEGG pathway enrichment analysis were performed. We defined the significant DEGs as those with *p* < 0.05, and the adjusted *p*‐value *q* < 0.1. The sequencing data of this study have been deposited in the NCBI SRA database (http://www.ncbi.nlm.nih.gov/sra) with the accession number: BioProject ID PRJNA751363.

### 4.14 Statistical analysis

The results were expressed as mean ± SD through at least three independent experiments, with *p *< 0.05 considered to be statistically significant. Comparison between two groups were analyzed by two‐tailed Student's *t* test and for multiple groups, the results were analyzed by one‐way analysis of variance (ANOVA). The results of survival comparison were analyzed by Kaplan–Meier survival curves and log‐rank test (Mantel–Cox method). All the statistical analyses were performed with GraphPad Prism 6.0 (GraphPad Software, CA, USA).

## CONFLICT OF INTEREST

The authors declare no conflict of interest.

## AUTHOR CONTRIBUTIONS

J‐M Dai and X‐Q Zhang were involved in the writing–original draft and editing, performed the preparation of the antibody; J‐J Zhang and W‐J Yang performed part of the in vitro and in vivo experiments; while X‐M Yang, H‐J Bian and Z‐N Chen were involved in the conceptualization, editing; and Z‐N Chen was involved in funding acquisition.

## ETHICS APPROVAL

The investigation was performed in accordance with the Regulations for the Administration of Affairs Concerning Experimental Animals approved by the State Council of People's Republic of China and was approved by the Ethical Committee for Animal Experimentation of Air Force Medical University (ID: IACUC‐20180128).

## Supporting information

Supporting Information

## Data Availability

The data generated and analyzed during the current investigation are available from the corresponding author on reasonable request.
