# Supplementary material for: Blockade of mIL‐6R alleviated lipopolysaccharide‐induced systemic inflammatory response syndrome by suppressing NF‐κB‐mediated Ccl2 expression and inflammasome activation
Source: MedComm (2020). 2022 May 6;3(2):e132. doi: 10.1002/mco2.132 (PMC9075038; doi:10.1002/mco2.132)
Supplement: Supplementary file 1 — Supporting Information [file MCO2-3-e132-s001.pdf]

Title page for Figure S1 and Table S1

Title:

Blockade of mIL-6R alleviated LPS-induced SIRS by suppressing NF-κB-mediated Ccl2 expression and inflammasome activation

Authors' names:

Ji-Min Dai[1,2#], Xue-Qin Zhang[1#], Jia-Jia Zhang[1], Wei-Jie Yang[1], Xiang-Min Yang[1*], Huijie Bian[1*], Zhi-Nan Chen[1*]

[#] These authors contributed equally to the manuscript.

Authors' affiliations:

[1] National Translational Science Center for Molecular Medicine＆Department of Cell Biology, State Key Laboratory of Cancer Biology, the Fourth Military Medical University, Xi'an, P.R. China.

[2] Faculty of Hepato-Biliary-Pancreatic Surgery, The First Medical Center of Chinese People's Liberation Army (PLA) General Hospital, Beijing, P.R. China.

*Correspondence's contact details:

X-M Yang, H-J Bian, & Z-N Chen.

National Translational Science Center for Molecular Medicine＆Department of Cell Biology, State Key Laboratory of Cancer Biology, the Fourth Military Medical University, 169 Changle West Road, Xi'an 710032, China.

Tel: +86-29-84773243; Fax: +86-29-83293906; E-mail: yxiangmind@163.com, hjbian@fmmu.edu.cn, znchen@fmmu.edu.cn

Key words: Ccl2, mIL-6R, monoclonal antibody, NF-κB, pyroptosis, SIRS

Running title: Blockade of mIL-6R alleviated LPS-induced SIRS

Type: Research article

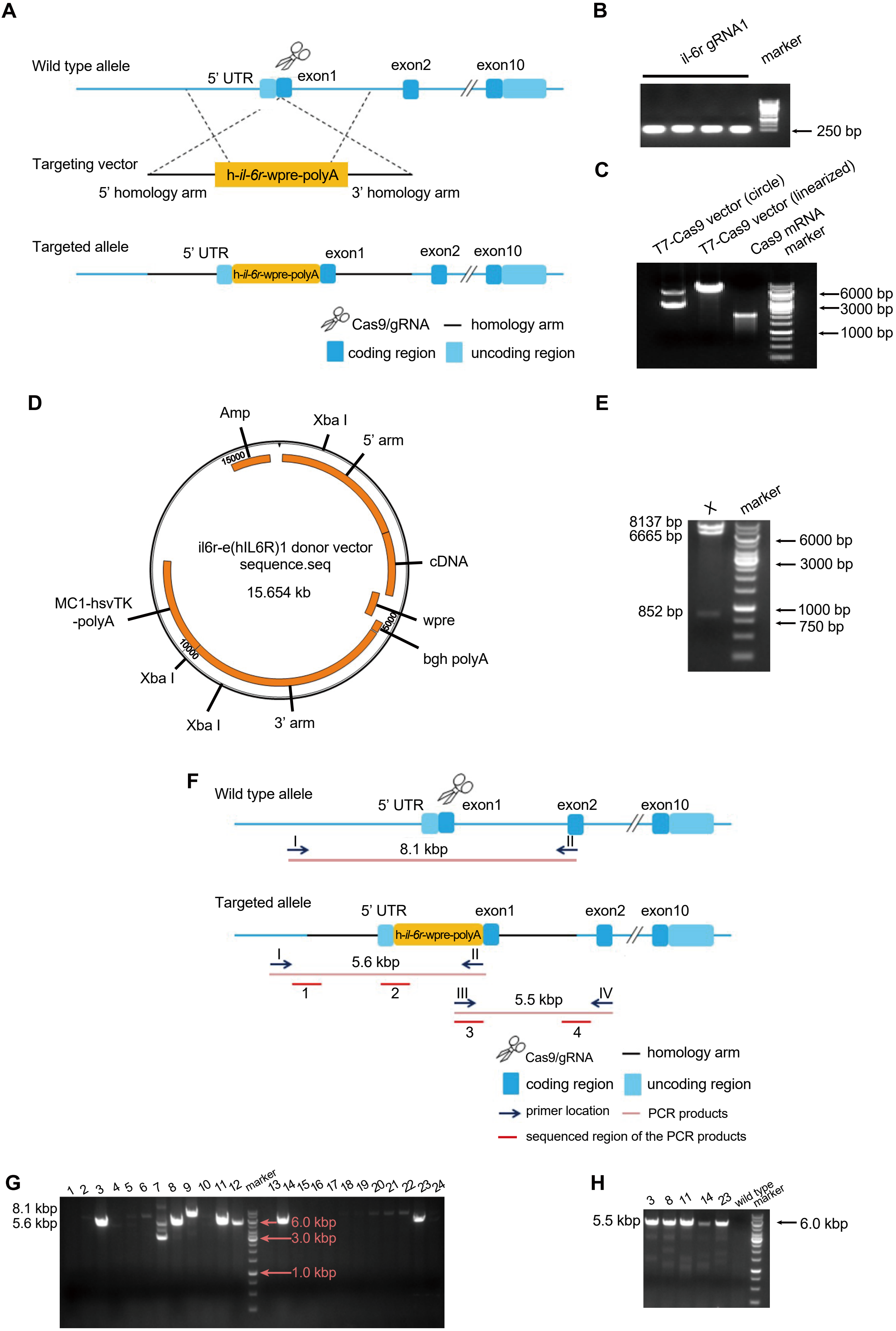
A
Wild type allele
5' UTR
exon1
exon2
exon10
Targeting vector
h-il-6r-wpre-polyA
5' homology arm
3' homology arm
Targeted allele
Cas9/gRNA
homology arm
coding region
uncoding region
B
il-6r gRNA1
marker
250 bp
C
T7-Cas9 vector (circle)
T7-Cas9 vector (linearized)
Cas9 mRNA
marker
6000 bp
3000 bp
1000 bp
D
Amp
Xba I
5' arm
cDNA
wpre
bgh polyA
3' arm
MC1-hsvTK
-polyA
15000
10000
5000
il6r-e(hIL6R)1 donor vector
sequence.seq
15.654 kb
E
X
marker
8137 bp
6665 bp
852 bp
6000 bp
3000 bp
1000 bp
750 bp
F
8.1 kbp
5.6 kbp
5.5 kbp
I
II
III
IV
1
2
3
4
primer location
PCR products
sequenced region of the PCR products
G
1 2 3 4 5 6 7 8 9 10 11 12 marker 13 14 15 16 17 18 19 20 21 22 23 24
8.1 kbp
5.6 kbp
6.0 kbp
3.0 kbp
1.0 kbp
H
3 8 11 14 23 wild type marker
5.5 kbp
6.0 kbp

Figure S1

The strategy and initial selection for generating and identifying of human *il-6r* gene knock-in mice.

(A) Schematic of the human *il-6r* gene knock-in location. wpre, the woodchuck hepatitis virus post-transcriptional regulatory element. (B,C) Nucleic acid electrophoresis results of the gRNA, T7-Cas9 (circle as well as linearized) vectors and Cas9 mRNA. (D) Schematic of homologous recombinant plasmid. (E) Nucleic acid electrophoresis result of the three fragments with the length of 852 bp, 6665 bp and 8137 bp spliced from the homologous recombinant plasmid, respectively. X represents that identification of the splicing sites was mediated by *Xba* I enzyme. (F) Schematic of identification for homologous recombination mice named IL6r-e(hIL6R)1. The product lengths of 5' and 3' homologous arm from wild type allele or targeted allele were 8.1 kbp, 0 kbp, 5.6 kbp and 5.5 kbp respectively. Four sequences shown with red line represented the sequenced region for PCR product identification. (G) Nucleic acid electrophoresis results of the 5' homology arm products from the target allele of the twenty-four P_0 transgenic mice. (H) Nucleic acid electrophoresis results of 3' homology arm products from the target allele of the indicated mice with the expected 5' homology arm products. All the experiments were conducted for three independent times.

Table S1. Sequence information for real time PCR primers used in this study

Gene	Species	Primers (5′→3′)	Product (bp)
actb	mouse	F[1]: GGCTGTATTCCCCTCCATCG<br>R[2]: CCAGTTGGTAACAATGCCATGT	154
il6ra	human	F: ATCGGGCTGAACGGTCAAAG<br>R: GGCGTCGTGGATGACACAG	77
cd33	mouse	F: GTTCCCTGTTCATCAGAGATGC<br>R: AGGAGTCCGTGATAGAGAGGT	137
ccl2	mouse	F: TTAAAAACCTGGATCGGAACCAA<br>R: GCATTAGCTTCAGATTTACGGGT	121

ccl7	mouse	F: GCTGCTTTCAGCATCCAAGTG<br>R: CCAGGGACACCGACTACTG	135
pigr	mouse	F: ATGAGGCTCTACTTGTTCACGC<br>R: CGCCTTCTATACTACTCACCTCC	100
naip1	mouse	F: TGCCCAGTATATCCAAGGCTAT<br>R: AGACGCTGTCGTTGCAGTAAG	116
hspa1b	mouse	F: GAGATCGACTCTCTGTTCGAGG<br>R: GCCCGTTGAAGAAGTCCTG	223
klrc3	mouse	F: TTAGCCTTCAACATGCTTCCC<br>R: TGGAAATGCAGGACACCAAAC	297

actb	human	F[1]: GGCTGTATTCCCCTCCATCG<br>R[2]: CCAGTTGGTAACAATGCCATGT	154
tnfa	human	F: CAGGCGGTGCCTATGTCTC<br>R: CGATCACCCCGAAGTTCAGTAG	89
il1b	human	F: CTGTGACTCATGGGATGATGATG<br>R: CGGAGCCTGTAGTGCAGTTG	75
il6	human	F: CTGCAAGAGACTTCCATCCAG<br>R: AGTGGTATAGACAGGTCTGTTGG	131
ccl2	human	F: CAGCCAGATGCAATCAATGCC<br>R: TGGAATCCTGAACCCACTTCT	190

[1]F (forward primer), [2]R (reverse primer)
